# Different meal, same flavor: cospeciation and host switching of haemosporidian parasites in some non-passerine birds

**DOI:** 10.1186/1756-3305-7-286

**Published:** 2014-06-23

**Authors:** Diego Santiago-Alarcon, Adriana Rodríguez-Ferraro, Patricia G Parker, Robert E Ricklefs

**Affiliations:** 1 Instituto de Ecología A.C., Biología y Conservación de Vertebrados, Carretera Antigua a Coatepec 351, El Haya. C.P., Veracruz, Xalapa 91070, Mexico; 2 Departamento de Estudios Ambientales, Universidad Simón Bolívar, Sartenejas, Venezuela; 3 Department of Biology, University of Missouri-St. Louis, One University Blvd., Saint Louis MO 63121, USA; 4 WildCare Institute, Saint Louis Zoo, One Government Dr., Saint Louis, MO 63110, USA

**Keywords:** Cospeciation, *Haemoproteus*, Haemosporida, Host switching, Non-passerines

## Abstract

**Background:**

Previous studies have shown that haemosporidian parasites (*Haemoproteus* (*Parahaemoproteus*) and *Plasmodium*) infecting passerine birds have an evolutionary history of host switching with little cospeciation, in particular at low taxonomic levels (e.g., below the family level), which is suggested as the main speciation mechanism of this group of parasites. Recent studies have characterized diverse clades of haemosporidian parasites (*H*. (*Haemoproteus*) and *H*. (*Parahaemoproteus*)) infecting non-passerine birds (e.g., Columbiformes, Pelecaniiformes). Here, we explore the cospeciation history of *H*. (*Haemoproteus*) and *H*. (*Parahaemoproteus*) parasites with their non-passerine hosts.

**Methods:**

We sequenced the mtDNA cyt b gene of both haemosporidian parasites and their avian non-passerine hosts. We built Bayesian phylogenetic hypotheses and created concensus phylograms that were subsequently used to conduct cospeciation analyses. We used both a global cospeciation test, PACo, and an event-cost algorithm implemented in CoRe-PA.

**Results:**

The global test suggests that *H*. (*Haemoproteus*) and *H*. (*Parahaemoproteus*) parasites have a diversification history dominated by cospeciation events particularly at the family level. Host-parasite links from the PACo analysis show that host switching events are common within families (i.e., among genera and among species within genera), and occasionally across different orders (e.g., Columbiformes to Pelecaniiformes). Event-cost analyses show that haemosporidian coevolutionary history is dominated by host switching and some codivergence, but with duplication events also present. Genetic lineages unique to raptor species (e.g., FALC11) commonly switch between Falconiformes and Strigiformes.

**Conclusions:**

Our results corroborate previous findings that have detected a global cospeciation signal at the family taxonomic level, and they also support a history of frequent switching closer to the tips of the host phylogeny, which seems to be the main diversification mechanism of haemosporidians. Such dynamic host-parasite associations are relevant to the epidemiology of emerging diseases because low parasite host specificity is a prerequisite for the emergence of novel diseases. The evidence on host distributions suggests that haemosporidian parasites have the potential to rapidly develop novel host-associations. This pattern has also been recorded in fish-monogenean interactions, suggesting a general diversification mechanism for parasites when host choice is not restricted by ecological barriers.

## Background

During the last decade, cophylogenetic studies on different groups of parasites have shown that the degree of association between host and parasite phylogenies depends on the nature of the interaction, including mode of transmission and dispersal capacity [1-5]. Although parasitologists long believed that avian haemosporidian parasites (including *Haemoproteus* (*Parahaemoproteus*) and *Plasmodium*) were host specific [6,7], recent studies have shown that haemosporidian parasites infecting passerine birds have an evolutionary history of host switching with little codivergence [2,8-12]. Nonetheless, host specificity at higher host taxonomic levels (e.g., families) has been observed for haemosporidian parasites of higher avian host taxa, such as the vireos (family Vireonidae) [13]. The low host specificity and high pathogenicity of some of these parasites has drawn attention in recent years to the study of avian haemosporidians, especially in light of other emerging infectious diseases that can spill over from bird hosts to novel hosts [e.g., [14-20].

The phylogenetic relationships among avian haemosporidians are reasonably well understood [21,22]. Recently, we have detected well-supported and highly diverse monophyletic clades of haemosporidians (e.g., *H*. (*Haemoproteus*)) that infect non-passerines [23-26]. These findings are in agreement with the traditional taxonomy of parasites infecting non-passerines [e.g., Columbiformes, [27-29]. In addition, a group of parasites of the subgenus *H*. (*Parahaemoproteus*) have been found to infect non-passerines, in particular raptors and sea birds [23,30,31]. However, cophylogenetic relationships between non-passerines and their *Haemoproteus* parasites are unexplored. In this study, we used mtDNA cyt b to analyze the cospeciation history of *H*. (*Haemoproteus*) and *H*. (*Parahaemoproteus*) with their non-passerine hosts. Following results of previous studies on passerine haemosporidians [2,9], we expected that *Haemoproteus* parasites infecting non-passerines are characterized by a cospeciation history at the family or higher taxonomic levels, and by a dynamic host switching pattern at the genus and species levels within families.

Most details of avian taxonomy are well established, but species concepts in the case of haemosporidian parasites are controversial [2,32,33], despite recent advances using *in vitro* hybridization experiments of different haemosporidian morphological species [e.g., [34]. Current species names for haemosporidian parasites are based primarily on morphological traits [27], but, in some cases, a variety of genetic lineages representing the same morphological species have been identified [e.g., [28,35]. Perkins [32] suggested that 3% mtDNA cyt b sequence divergence or more would be indicative of different morphological species based on her studies of lizard *Plasmodium*. Subsequently, Hellgren *et al.*[35] suggested that a genetic difference larger than 5% in the cyt b gene corresponds to morphologically differentiated haemosporidian parasites [e.g., [28]. However, this rule is not supported in the opposite direction, where morphologically differentiated haemosporidians occur with genetic differences less than 5% [36,37]. Bensch *et al.*[33] used nuclear and mtDNA loci and proposed that haplotypes identified by the use of cyt b might represent reproductively isolated entities. Their conclusion was based on the significant statistical association or parallel evolution of both nuclear and mitochondrial genes and on linkage disequilibrium between lineages, even when their hosts were sympatric [33]. Hence, because morphological species can represent a diversity of independently evolving genetic lineages, we regard haemosporidian mtDNA cyt b lineages as independently evolving entities, or species, in this study, recognizing that some closely related lineages might represent genetic variation within species [38].

## Methods

We used mtDNA cyt b sequences of haemosporidian parasites (subgenus *Haemoproteus* and *Parahaemoproteus*) from previous studies [23-26,30,31,39] that are either attached to a morphologically identified species or that are haplotypes belonging to a well supported phylogenetic clade (e.g. lineage GQ395631). We sequenced the mtDNA cyt b for avian hosts that were not available from GenBank™ and for which we had blood samples, using primers L14841 [40] and H4a [41] to amplify ca. 1045 bp of mtDNA cyt b of bird hosts. PCR conditions were as described in [42]. When several bands were present in the amplified products, we optimized the reaction and then purified the targeted fragment by using QIAquick gel extraction kit (QIAGEN). If a single band was obtained, we cleaned the PCR product using Antarctic phosphatase and Exonuclease I (#M0289S and #M0293S respectively, New England Bio Labs, Inc.). We either used an ABI 3100 microcapillary genetic analyzer to sequence DNA products or sent the samples for sequencing at Macrogen, Inc. (Seoul, Korea). When host mtDNA cyt b sequences were not available from GenBank™ and we did not have a DNA sample, we substituted a sequence of a closely related species from the same genus (e.g. *Alcedo atthis* substituted *A. leucogaster*, *Strix butleri* substituted *S. seloputo*).

Sequences were edited in SeqManII version 4 (1989–1999. DNASTAR, Inc.) and aligned by eye in Se-Al v2.0a11 (1996–2002, http://tree.bio.ed.ac.uk/software/seal/). Phylogenetic hypotheses were constructed using the program MrBayes v3.1.2 [43]. Two independent runs were made, with 4 chains in each run for a total of 1 million generations, sampling every 100 generations. The first 5000 trees (50%) were discarded as the ‘burn-in’. In total, 5000 trees from each run were used to build our majority-rule consensus tree. A TIM2 + G model of molecular evolution was used for parasites with shape parameter α = 0.26, and a TPM3uf + I + G model for hosts with shape parameter α = 0.75 and proportion of invariable sites Pinvar = 0.47, as suggested by jModelTest v2.1.2 [44,45]. To implement the two models in Mr. Bayes we used the two parameter model (Nst = 2) for parasites and the next more complex available model (GTR, Nst = 6) for hosts, as recommended in the user’s manual; finally, we fixed the parameters (gamma shape parameter, proportion of invariable sites, nucleotide frequencies, nucleotide substitution rates) according to the values calculated with jModelTest for each case. Parasite trees were rooted using a lineage (accession number: JX993047) from the *Plasmodium relictum* morphospecies. Although some phylogenetic reconstructions have shown a paraphyletic relationship between the subgenera *Haemoproteus* and *Parahaemoproteus*[22,23], more recent analyses have shown that the two *Haemoproteus* subgenera are reciprocally monophyletic and sister to *Plasmodium* parasites [28,46,47], validating our outgroup selection. The host tree was rooted using a sequence (accession number: AY283492) from *Amazona albifrons* (Psittaciformes), and phylogenetic relationships of hosts were verified following [48]. New sequences were deposited in GenBank™ for both parasites [GenBank: KF924042] and birds [GenBank: KF924043, KF924044, KF924045, KF924046].

For cospeciation analyses, we first used PACo, a global test implemented in R [49] that uses an approach similar to ParaFit [50]. This test was used to quantitatively assess the congruence between two phylogenies, and to identify the associations (i.e., host-parasite links) contributing to the cophylogenetic structure [49]. PACo requires three matrices, one where host (rows) – parasite (columns) associations are indicated as presence/absence (host-parasite links), and two genetic distance matrices, which are calculated from the host and parasite phylogenetic trees. The host-parasite link matrix is transformed into an identity matrix to accommodate multiple associations (e.g., hosts harboring more than one parasite species/lineage and vice versa) and the two distance matrices are transformed into matrices of principal coordinates (PCo). Next, Procrustes analysis is applied using least-squares superimposition to yield a residual sum of squares *m*^*2*^_*XY*_ (i.e., to determine the fit of the parasite PCo onto the host PCo), which is inversely proportional to the topological congruence between the parasite and host PCos. Hence, *m*^*2*^_*XY*_ is a measure of the fit of the parasite phylogeny onto the host phylogeny, providing a goodness-of-fit statistic whose significance is established by a randomization procedure. In our case, significance was assessed comparing the observed *m*^*2*^_*XY*_ to those generated by 10,000 random permutations (*m*^*2*^_XY_*) of the host-parasite association matrix (hosts were randomly allocated to parasites). PACo then tests the null hypothesis (H_0_) that parasite clades are randomly associated with host clades: the one-tailed probability *P* is the proportion of *m*^*2*^_XY_* values ≤ *m*^*2*^_*XY*_. We also assessed the contribution of individual host-parasite associations to the global fit using the fact that *m*^*2*^_*XY*_ is the sum of squared residuals of each host-parasite link *e*_*i*_^*2*^, which provides a direct measure of the importance of each link. The *e*_*i*_^*2*^’s and their 95% confidence intervals were estimated using a jackknife method. Each *i*^*th*^ link is replaced by 0 (i.e., we eliminate that specific host-parasite association from our matrix), then we estimate the *e*_*i*_^*2*^ squared residual for each host-parasite link to assess its contribution to the global cospeciation fit; and those links that contribute relatively little to *m*^*2*^_*XY*_ likely represent coevolutionary links [see [49] for more details].

We then used CoRe-PA v0.5.1 [51], which performs an event-cost-based analysis. Such an approach aimed to find the most probable coevolutionary history (i.e., all feasible solutions to the host-parasite reconciliation problem) of the associated taxa using the parsimony criterion (i.e., lowest cost), via exhaustive searches based on an event-cost scheme. Unlike global tests, event-cost methods try to identify the type of evolutionary events (i.e., cospeciation, sorting, duplication, and switching) explaining the most parsimonious solutions. We used 100 randomizations of host-parasite associations to determine whether the number of each event type differed significantly from random associations between the two trees [51]. We used an event-cost scheme similar to previous studies to make our results comparable (Table 1). Also, because most event-cost software programs are designed to maximize the number of cospeciation events, we set the cost for codivergence low and then implemented different costs for the other three evolutionary processes (duplication, sorting, switching) implemented in the program to determine their relative contributions. These algorithms can find more than one optimal solution for the same event-cost scheme, in which case we present results for the solutions that maximize both the number of cospeciation events and the number of host switches. Host switches are expected to be more common among closely related hosts due to similarities in physiological traits [e.g., immunology, [15], but most parasite switches do not result in successful infections and novel hosts are commonly dead-end organisms for parasites [52]. Accordingly, we implemented a cost scheme for host switching events decreasing from difficult (cost = 3) to easy (cost = 0).

**Table 1:** Cost-event codivergence analysis for the distribution of mtDNA cyt b lineages of haemosporidian parasites across their avian hosts

**Event costs**^**a**^				**Total cost**	**Codivergence**	**Duplication**	**Sorting**	**Switching**
1	1	0	1	33	0^c^	29	91^b^	0^c^
0	2	1	3	57	11	4-5	7-8^b^	13^c^-14
0	0	1	2	34	8^b^-11^b^	5-7	4-8^b^	13^c^-15
1	1	1	1	29	0-6^b^	1^c^-8^b^	0	19-28^b^
1	1	1	0	1	0	1	0	28

For each analysis we conducted 100 randomizations to determine whether the predicted number of each event type is higher or lower than expected by chance.

^a^Event costs are for codivergence (cospeciation), duplication (within-host speciation), sorting (extinction), and host switching, respectively.

^b^The number of events significantly exceeds that for randomized trees (P < 0.05).

^c^The number of events is significantly less than that for randomized trees (P < 0.05).

## Results

**Figure 1:**
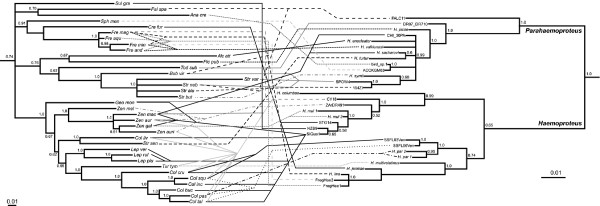
**Tanglegram showing the two majority-rule consensus phylograms for the associations between haemosporidian parasites and non-passerine hosts.** Bayesian posterior probability is shown for each node above branches. Different line types were used as a visual aid to tease apart host-parasite associations. Host species names: *Cre fur = Creagrus furcatus* (H1)[EF373127]*, Fre mag = Fregata magnificens* (H2)[FR691291]*, Fre min = Fregata minor* (H3)[JX569167]*, Fre and = Fregata andrewsi* (H4)[JX416759], *Fre aqu = Fregata aquila* (H5)[EU166990], *Col pas* = *Columbina passerina* (H6)[KF924046], *Col tal* = *C. talpacoti* (H7)[KF924043], *Col buc* = *C. buckleyi* (H8)[KF924044], *Col squ* = *Columbina squammata* (H9)[AF483347], *Col inc* = *C. inca* (H10)[AF182683], *Col cru* = *C. cruziana* (H11)[KF924045], *Tur tym = Turtur tympanistria* (H12)[HM746793]*, Lep ruf* = *Leptotila rufaxilla* (H13)[AF182698]*, Lep plu* = *L. plumbeiceps* (H14)[AF279707], *Lep ver* = *L. verreauxi* (H15)[AF279705], *Col liv* = *Columba livia* (H16)[AF182694], *Str sen = Streptopelia senegalensis* (H17)[AF279710], *Zen mac* = *Z. macroura* (H18)[AF182703], *Zen aur* = *Z. auriculata* (H19)[AF182700], *Zen gal* = *Z. galapagoensis* (H20)[AF182701], *Zen auri* = *Z. aurita* (H21)[AF182704], *Zen mel* = *Zenaida meloda* (H22)[AF182699], *Geo mon* = *Geotrygon montana* (H23)[AF182696], *Str var = Strix varia* (H24)[AF448260]*, Str neb = S. nebulosa* (H25)[AJ004059], *Str alu = Strix aluco* (H26)[AY422982], *Str but = Strix butleri* (H27)[EU348994], *Bub vir = Bubo virginianus* (H28)[AF168106]*, Tod sub = Todus subulatus* (H29)[HM222445]*, Alc att = Alcedo atthis* (H30)[D38329], *Pic pub = Picoides pubescens* (H31)[DQ479260], *Fal spa = Falco sparverius* (H32)[NC_008547]*, Ana cre = Anas crecca* (H33)[EU585607], *Sul gra = Sula granti* (H35)[JX569182], *Sph men = Spheniscus mendiculus* (H36)[DQ137219]. Parasite species names: *H. jen = Haemoproteus jeniae* (P1)[JN827320]*, H. iwa* = *Haemoproteus iwa* (P2)[JF833050], FregHae2 = *Haemoproteus* lineage FregHae2 (P3)[HQ400763], FregHae1 = *Haemoproteus iwa* lineage FregHae1 (P4)[HQ400760], *H. mul* 1 = *Haemoproteus multipigmentatus* (P5)[GU296220], STG14 *= Haemoproteus* lineage STG14 (P6)[JF833066], NZB9 = *Haemoproteus* lineage NZB9 (P7)[JF833059], SIGua1 = *Haemoproteus multipigmentatus* (P8)[FJ462680], *H. mul* 2 = *Haemoproteus multipigmentatus* (P9)[GU296222], AZrDR491 = *Haemoproteus* lineage AZrDR491 (P10)[FJ462650], CY18 = *Haemoproteus* lineage CY18 (P11)[JF833042], *H. columbae* = *Haemoproteus columbae* (P12)[EU254549], FALC11 = *H.* (*Parahaemoproteus*) lineage FALC11 (P14)[GQ141558], SPOW4 = *H.* (*Parahaemoproteus*) lineage SPOW4 (P15)[EU627833], 154ZI = *H.* (*Parahaemoproteus*) lineage 154ZI (P16)[KF279523], *H. syrnii = H.* (*Parahaemoproteus*) *syrnii* (P17)[DQ451424], CHI_30PA = *H.* (*Parahaemoproteus*) lineage CHI_30PA (P18)[KF924042], *H. sacharovi = H.* (*Parahaemoproteus*) *sacharovi* (P19)[JX073258], *H. turtur = H.* (*Parahaemoproteus*) *turtur* (P20)[DQ451425], *H. enucleator = H.* (*Parahaemoproteus*) *enucleator* (P21)[DQ659592], bird_sp.1 = *H.* (*Parahaemoproteus*) lineage bird_sp.1 (P22)[GQ141557], ACCKGM63 = *H.* (*Parahaemoproteus*) lineage ACCKGM63 (P23)[GQ395631], *H. valkiunasi = H.* (*Parahaemoproteus*) *valkiunasi* (P24)[GQ404559], *H. picae = H.* (*Parahaemoproteus*) *picae* (P25)[EU254552], DR07_DR710 = *H.* (*Parahaemoproteus*) lineage DR07_DR710 (P26)[HM222464], SSFL03Ven = *Haemoproteus* lineage SSFL03Ven (P27)[FJ462661], SSFL06Ven = *H.* lineage SSFL06Ven (P28)[FJ462663], *H. par.* 2 = *Haemoproteus paramultipigmentatus* (P29)[JN788939], *H. par* 1 = *Haemoproteus paramultipigmentatus* (P30)[JN788934], *H. multivolutinus* = *Haemoproteus multivolutinus* (P31)[JX275888]. Parentheses enclose the code numbers used in Figure 2 for both hosts and parasites, and squared brackets enclose the GenBank™ accession numbers.

Parasite phylogenies were obtained in previous studies [23-25]. Here we used pruned versions of those phylogenetic trees for cospeciation analyses; consensus phylograms were calculated using unique haplotypes from well-supported clades. The avian host phylogeny was well supported and is in agreement with the phylogenomic study of birds from [48]. Relationships among Columbiformes followed that suggested by [53]. Consensus phylograms for both parasites and hosts are presented along with their tanglegram depicting host-parasite associations (Figure 1).

**Figure 2:**
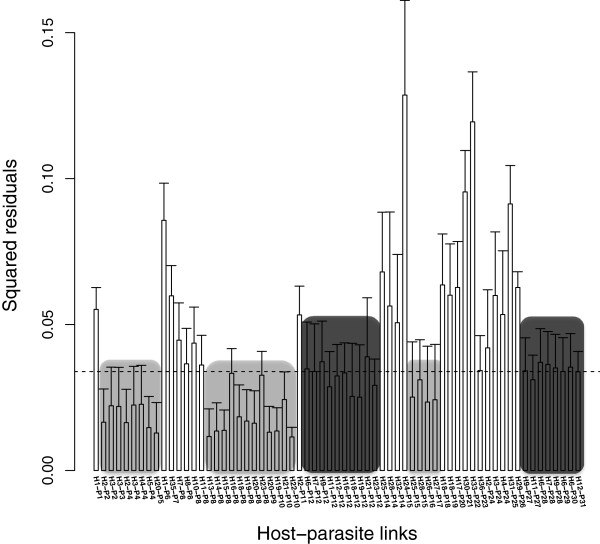
**Contributions of individual host-parasite links to the global cospeciation fit.** Jackknifed squared residuals (bars) and upper 95% confidence intervals (error bars) resulting from applying PACo to genetic distances derived from phylogenetic trees presented in Figure 1. The median squared residual value is shown (dashed line) to facilitate comparisons among different host-parasite links. Host-parasite links with low squared residual (*e*^*2*^_*i*_) value likely represent coevolutionary links (see Methods). Light gray boxes indicate host-parasite links with a more important contribution to the global codivergence signal in comparison to those links enclosed by dark boxes. See Figure 1 for host-parasite links code numbers.

We detected a significant global signal of cospeciation between *Haemoproteus* (*Haemoproteus* and *Parahaemoproteus*) parasites and their non-passerine hosts (*m*^*2*^_*XY*_ = 2.3, *P* < < 0.0001). The global cospeciation signal is mostly due to three groups: 1) host-parasite links involving pigeons and doves infected by several lineages of the morphospecies *H. multipigmentatus*, 2) host-parasite links involving frigate birds infected by lineages of the morphospecies *H. iwa*, and 3) host-parasite links involving raptors from the genera *Strix* and *Bubo* infected with lineages *H.* (*Parahaemoproteus*) SPOW4 and *H*. (*Parahaemoproteus*) *syrnii* (see light gray boxes in Figure 2). A second group contributing to the significant global cospeciation signal, albeit to a lesser degree (i.e. cospeciation patterns are less congruent compared to links in light gray boxes), includes pigeon and doves infected with lineages of *H. columbae*, *H. paramultipigmentatus*, *H. multipigmentatus*, and *H. multivolutinus* (dark gray boxes in Figure 2). Host-parasite links show that host switching is frequent within families (i.e., among genera and among species within genera), and occasionally across different orders (e.g., Columbiformes to Pelecaniiformes, Falconiformes to Strigiformes Figures 1 and 2).

Event-cost analyses showed that statistically well-supported event combinations ranged between 29 and 57 events total cost (Table 1). The events suggest an evolutionary history dominated by host switching and some codivergence, with a lesser influence of duplication events (Table 1, event-costs 0012 and 1111). Even when we make host switching a costly event (event-costs 0213 and 0012), our analysis identified host switching as a prominent mechanism in the evolutionary history of haemosporidians.

Among the novel relationships identified in this analysis, parasite lineage CHI_30PA, which infects a *Zenaida macroura* dove, belongs to the well suppoted *H*. (*Parahaemoproteus*) subgenus (Figure 1). We conducted a sequence similarity analysis (BLAST algorithm of the NCBI database) between our lineage CHI_30PA and those identified by [47]. We found that our *Z. macroura* dove parasite lineage (sample from Chicago, USA) is 99% similar to several lineages infecting raptors sampled in California (e.g. GenBank EU627839 and EU627842). The *H. turtur* lineage infecting *Streptopelia* doves identified by [47] is sister to the *H. sacharovi* lineage infecting *Z. macroura* doves (Figure 1). All other parasites infecting pigeons and doves included in this analysis belong to the subgenus *Haemoproteus* (Figure 1).

## Discussion

Ricklefs and Fallon [9] found that closely related haemosporidian parasites are conservatively distributed within host higher taxa, suggesting that codivergence or switching among closely related hosts is a common event in their evolutionary history [2,54]. In addition, duplication (within host speciation) events were modeled by TreeFitter as being frequent among these parasites when the event cost was set low [2]. By using the same gene (mtDNA cyt b) from previous studies, we have found that host switching is a commonly observed mechanism between haemosporidians and their non-passerine hosts, mainly close to the tips of the host phylogenetic tree (i.e., between close relatives). Codivergence is also observed in the evolutionary history of haemosporidian parasites infecting non-passerine birds, in particular at the family level (Figure 1). Duplication (within-host parasite lineage splitting) has also played some role in the diversification of non-passerine Haemosporida, which was also recognized in haemosporidian parasites infecting passerine birds under low event costs [2]. Even when codivergence is a prominent feature of our system, strict host specificity at the species level is rare. These findings suggest a dynamic recent evolutionary history between haemosporidians and their bird hosts, which imply that host switching, in combination with geographic isolation, might be an important mechanism in the formation of new haemosporidian species (Ricklefs *et al.*, unpublished). Cospeciation studies in fish-monogenean systems have also identified host switching as an important diversification mechanism aided by subsequent host isolation, producing a higher degree of specificity at the family level or above [55,56]. This suggests that the evolutionary history of these parasite systems is dominated by traits (e.g., immunology of hosts) that are conserved at high taxonomic levels (i.e., family or above). Where ecological barriers do not prevent parasites from switching hosts, related parasite lineages can infect distantly related hosts, as in the case of closely related *Haemoproteus* parasite lineages (*H. multipigmentatus*, STG14, NZB9) infecting distantly related sea birds (e.g., *Sula granti*, *Creagus furcatus*) and doves (e.g. *Zenaida galapagoensis*), which occur on the same islands [25]. In the absence of such opportunities, resulting from behavioral or geographic host isolation, co-speciation might become more likely [see [55,56] for the case of fish-monogenean systems].

It is important to consider that we are leaving aside the vectors (Diptera) in this analysis of host-parasite interactions. How do haemosporidians relate coevolutionarily to dipteran vectors? It is a question for which we have almost no insight. Previous work suggests that different dipteran families are specialized in transmitting different Haemosporida genera [e.g., Ceratopogonidae transmit only *Haemoproteus* (*Parahaemoproteus*) parasites, [22,47]. Recent work on Culicidae [57-59] and Ceratopogonidae [18,19,60,61] shows that many vector species have broad feeding preferences, even across vertebrate classes. Thus, vectors may come in contact with a diverse array of Haemosporida parasites, including genera they do not normally transmit [e.g., [18,60,62-64]. The fact that many vector species have broad vertebrate host preferences and are susceptible to infections by different Haemosporida genera, would suggest that specificities of both vertebrate and insect host immune systems would mediate parasite “jumps” across distantly related avian hosts [see [15,64]. Experimental studies have demonstrated that an avian parasite, *Plasmodium lophurae*, can adapt to and be viable in mice after just four rounds of infectious inoculations [65]. Furthermore, erythrocytes of different mammal species have been shown to be susceptible to invasion by bird *Plasmodium* parasites [66]. Hence, these studies suggest that haemosporidian parasites could potentially adapt to phylogenetically distant hosts.

Parasites of the sub-genus *Haemoproteus*, which are normally transmitted by louse flies (Diptera: Hippoboscidae), might be less likely to switch between unrelated hosts because normally hippoboscid flies are host specific and do not fly long distances [67]. However, recent work in the Galapagos Islands has shown that lineages belonging to the same morphospecies (*H. multipigmentatus*) infect endemic doves [24,28] and sea birds inhabiting the same islands [frigate birds, gulls, brown boobies, [25], indicating host sharing by the flies even though different-sized hippoboscid species are associated with these different bird species. Furthermore, hippoboscid flies (*Olfersia spinifera* and *O. aenescens*) have higher rates of gene flow in comparison the their seabird hosts, which might explain the lack of genetic structure of the *Haemoproteus iwa* parasite across the Galápagos Islands [68]. Finally, the evolutionary history of the Hippoboscidae includes at least two host switches from mammals to birds [67], suggesting that parasites either hitchhiking with louse flies [e.g. phoretic mites, [69] or developing within them (e.g. *Haemoproteus*) can “jump” across large host phylogenetic distances. Hence, under the right ecological conditions (e.g. simple systems like island faunas, different bat species sharing roosts [70], similar parental care behaviour across different cichlid species [71]) parasites, in particular haemosporidians transmitted by hippoboscid flies, are capable of switching across distantly related hosts (see Figure 1).

Our vertebrate host sample includes species that belong to the same genera (e.g., *Columbina* spp., *Zenaida* spp.) within the same host sub-family (e.g., Columbidae: Columbinae). The distribution of parasites among host species reported here supports a degree of host specificity below the family level for parasite lineages (e.g., lineages of *H. multipigmentatus*, *H. iwa*, *H*. (*Parahaemoproteus*) *syrnii*, see Figure 1), which is validated by the cospeciation events and the global cospeciation signal detected in our system. However, that parasite haplotypes of *H*. (*Haemoproteus*) infect many species and genera within Columbiformes [24], sea birds of the same genus e.g., *Fregata*, [25], and host species across orders (e.g., *H*. (*Parahaemoproteus*) FALC11, Figure 1), lends support to a diversification history dominated by host switching. Our sample consists mostly of a small number of non-passerine species of the sub-family Columbinae, the genera *Fregata*, *Creagus*, *Todus*, *Falco*, *Bubo*, *Strix*, *Anas*, *Spheniscus*, *Picoides*, and *Alcedo* along with their described haemosporidian parasites, which contrasts with analyses of widely sampled passerine hosts and their parasites [see [2,9]. Thus, although our results support previous findings, a broader non-passerine taxonomic sampling with broader geographical distribution will be required to produce a more comprehensive understanding of haemosporidian diversification.

Previous work considered that all parasites infecting Columbiformes belonged to the *H*. (*Haemoproteus*) subgenus [24,28]. However, in this study we identified a parasite lineage (CHI_30PA) from a *Zenaida macroura* dove that falls within the strongly supported *H*. (*Parahaemoproteus*) subgenus, which is in agreement with other recent examples of columbiform parasites (*H. sacharovi* and *H. turtur*) that belong to the subgenus *Parahaemoproteus* and likely are transmitted by *Culicoides* vectors [47].

## Conclusion

In summary, our study shows that the codivergence history of haemosporidian parasites with their avian hosts is dominated by host switching events; cospeciation is mostly observed at the family or higher taxonomic levels [see also [2,9]. Such dynamic host-parasite associations are relevant to the epidemiology of emerging diseases. Low parasite host specificity (i.e., as shown by generalist and widely distributed parasites) is a prerequisite for the emergence of new diseases [72], and it has been observed that generalist malaria parasites reach higher prevalence than specialist parasites in the local populations they infect [e.g., [73,74]. Moreover, phylogenetically related haemosporidian lineages (e.g., sister lineages) do not necessarily have a similar host breadth and geographic distribution [74], which challenges our ability to predict novel haemosporidian zoonoses. Taken together, the evidence on host distributions suggests that haemosporidian parasites have the potential to rapidly develop novel host-associations [19], but see [64].

## Competing interests

The authors declare that they have no competing interests.

## Authors’ contributions

Conceived and designed the study: DS-A, PGP, RER. Contributed reagents/material/analysis tools: PGP, AR-F, DS-A. Wrote and revised critically the manuscript: DS-A, AR-F, PGP, RER. All authors have read and approved the final manuscript.
